# Effects of *Rhodopseudomonas palustris* on the Rumen Microbiota of Leizhou Goats

**DOI:** 10.3390/ani15233390

**Published:** 2025-11-24

**Authors:** Longqing Zheng, Danju Kang, Xuanhui He, Fuquan Yin, Shangquan Gan, Guangxian Zhou

**Affiliations:** College of Coastal Agricultural Sciences, Guangdong Ocean University, Zhanjiang 524088, China; 17309407272@stu.gdou.edu.cn (L.Z.); kangdj@gdou.edu.cn (D.K.); hxh04103@163.com (X.H.); yinfq@gdou.edu.cn (F.Y.); shangquangan@gdou.edu.cn (S.G.)

**Keywords:** Leizhou goat, *Rhodopseudomonas palustris*, rumen microbiota, full-length 16S rRNA gene sequencing

## Abstract

This study investigated the effects of *Rhodopseudomonas palustris* (*R. palustris*) supplementation on the rumen microbiota of Leizhou goats and explored its potential mechanisms. Thirty healthy Leizhou goats of similar weight and age were selected and randomly assigned to five groups (six goats per group) using a completely randomized block design for a 75-day feeding trial. The control group (CONRF) was fed a basal diet, and the Photosynthetic Bacteria Medium (PBMRF) group was fed a basal diet + PBM solution. The low-concentration *R. palustris* (LRPRF), medium-concentration *R. palustris* (MRPRF), and high-concentration *R. palustris* (HRPRF) groups were fed a base diet supplemented with 20.0 mL, 40.0 mL, and 80.0 mL of *R. palustris* solution, respectively. All supplements were administered by mixing them into the feed. On day 75 of the trial, three goats were randomly selected from each group for slaughter and evisceration. Rumen contents were collected, immediately filtered, aliquoted, quick-frozen in liquid nitrogen, and stored at −80 °C for subsequent analysis of rumen microbial diversity. Rumen microbial community structure was analyzed using high-throughput sequencing. The results showed that *R. palustris* enriched unique operational taxonomic units (OTUs), particularly in the LRPRF group. At the phylum level, Firmicutes and Bacteroidota were dominant; Firmicutes and Euryarchaeota abundance increased, while Bacteroidota decreased in the experimental groups. In addition, Verrucomicrobiota abundance was significantly elevated (*p* < 0.05). At the genus level, Prevotella was predominant, whereas *Selenomonas* abundance was significantly reduced (*p* < 0.01). Meanwhile, compared to the CONRF, PBMRF, and LRPRF groups, the MRPRF and HRPRF groups exhibited higher relative abundances of *Christensenellaceae_R-7 group* and *Anaeroplasma*. LEfSe analysis revealed a greater number of differential taxa in the experimental groups compared with the control, including enrichment of beneficial bacteria, such as *Lactobacillus*, which may contribute to optimizing the rumen environment by regulating immune and metabolic functions. Functional prediction indicated that rumen microorganisms were mainly involved in carbohydrate and amino acid metabolism. In conclusion, supplementation with *R. palustris* can beneficially modulate rumen microbial composition and function and promote rumen absorption of nutrients and degradation of crude fiber. This study provides a theoretical basis for green goat farming practices.

## Simple Summary

*Rhodopseudomonas palustris* (*R. palustris*) is a nutrient-rich bacterium with a protein content of up to 65% and abundant bioactive compounds. In this study, we evaluated its effects on the rumen microbiota of Leizhou goats. A 75-day feeding trial combined with high-throughput sequencing demonstrated that it increased the number of unique operational taxonomic units (OTUs), with the most pronounced effect observed in the low-concentration *R. palustris* (LRPRF) group. At the phylum level, the relative abundance of the Verrucomicrobiota community in the *R. palustris* group was significantly higher than that in the CONRF and PBMRF groups (*p* < 0.05). Compared with the CONRF group, the relative abundance of the Firmicutes and Euryarchaeota showed an upward trend, while that of the phylum Bacteroidota exhibited a downward trend. At the genus level, *Prevotella* remained dominant, whereas Methanobrevibacter abundance declined, and several probiotic taxa were significantly enriched. Functional prediction indicated that the rumen microbiota was primarily associated with carbohydrate and amino acid metabolism. These findings suggest that *R. palustris* supplementation can beneficially modulate the rumen microbial community, promote the absorption of nutrients, such as carbohydrates, and the degradation of coarse fibers, like lignocellulose, in the rumen, thereby enhancing the rumen digestive function of Leizhou goats while providing a theoretical basis for ecological and sustainable farming.

## 1. Introduction

The rumen, as a large and complex fermenter in ruminants, has a microbial community that is the core support of ruminant life activities and plays a decisive role in the enhancement of animal health and production performance [1,2]. The rumen is inhabited by trillions of microorganisms such as bacteria, fungi, protozoa, and archaea [3]. They form a highly synergistic ecosystem. These microorganisms can take plant polysaccharides, among other structures, such as cellulose and hemicellulose, which are indigestible by ruminants themselves [4,5] and are efficiently degraded to volatile fatty acids, among other metabolites and byproducts (NH_3_, CO_2_, CH_4_, H), providing over 70% of the host’s energy source [6,7]. At the same time, microbial industrial fermentation produces mycoprotein, which also becomes a high-quality protein supplement for animals [8]. In addition, rumen microorganisms are also involved in regulating the development of the host immune system, and through interactions with intestinal mucosal immune cells, they enhance animal resistance to pathogenic bacteria and maintain healthy homeostasis in the body [9,10,11]. Therefore, in-depth exploration of the functional potential of rumen microorganisms and precise regulation of their flora structure has become a research hotspot in the field of ruminant nutrition and healthy breeding.

*R. palustris*, a purple non-sulfur bacterium with metabolic diversity, carbon source diversity, and metabolite diversity, has attracted attention for its unique metabolic properties and beneficial functions as a photosynthetic bacterium widely found in nature [12]. The bacterium can utilize a variety of carbon and nitrogen sources for growth and reproduction and can fix nitrogen, degrade harmful substances, and synthesize vitamins [13,14,15]. It demonstrates good potential for applications in aquaculture, wastewater treatment, and livestock rearing [15,16,17,18]. Studies have shown that *R. palustris* can secrete a variety of digestive enzymes, such as cellulase and protease, which are directly involved in the degradation process of feed and accelerate the release of nutrients [19]. At the same time, its metabolism produces vitamins, amino acids, and other active substances, which can enhance the antioxidant capacity of the animal body and improve immunity [20,21]. In livestock and poultry breeding, *R. palustris* can promote animal growth and development and improve feed utilization by regulating the structure of intestinal flora, enhancing the antioxidant capacity and immune function of the body [22,23]. Studies on rumen digestion and fermentation in cows have revealed that *R. palustris* demonstrates significant potential in promoting rumen microbial growth and promoting microbial fermentation for non-sugar energy supply. This process relies on the maintenance of an anaerobic environment, a prerequisite for microbial homeostasis [24].

As a characteristic local breed in Guangdong Province, the Leizhou goat occupies an important position in regional economic development [25]. However, in the process of large-scale farming, problems such as low feed conversion efficiency, high farming costs, and unstable product quality have gradually come to the forefront [26]. Given the potential advantages demonstrated by *R. palustris* in in vitro rumen studies of ruminants and the underdeveloped research field regarding its effects on the goat rumen microbiota, this study selected geographically representative Leizhou goats as subjects to investigate the effects of *R. palustris* on the rumen microbial diversity of Leizhou goats, aiming to elucidate its mechanisms in improving microecological balance, stabilizing pH levels, alleviating acidosis, and modulating the gut microbiota by augmenting beneficial populations and restricting pathogenic ones. These findings provide theoretical foundations and technical support for optimizing Leizhou goat farming practices and advancing green, efficient livestock development.

## 2. Materials and Methods

### 2.1. Animal Ethics Statement

The Animal Care Facility and Ethics Committee of Guangdong Ocean University approved this experiment. All animal experiments were approved by the Animal Ethics Committee of Guangdong Ocean University, with permit number GDOU (Guangdong)-2024N0930-23 (dated 30 September 2024).

### 2.2. Design and Housing

The experimental animals were provided by the Leizhou Goat Conservation Farm—Guangdong Ocean University Branch. Thirty healthy Leizhou goats were selected, all with similar body weights (approximately 16.0 ± 2.0 kg) and consistent ages (approximately 8–10 months). After a 5-day adaptation period, they were randomly assigned to 5 treatment groups. Each treatment group comprised six goats. All goats were housed in pens (3.5 m long × 5 m wide), each equipped with feeders and drinking troughs supplying fresh water. Each pen housed six goats to allow free movement. A 5-day adaptation period was provided during which pens were disinfected and goats underwent deworming, and they became familiar with the experimental environment, including feeders, basal diet, and waterers. After the 5-day adaptation period, goats’ health status and environmental suitability were carefully assessed, and the feeding trial commenced. The control group (CONRF) goats were fed a basal diet. The Photosynthetic Bacteria Medium (PBMRF) group received the basal diet supplemented with PBM solution. The low-concentration *R. palustris* (LRPRF), medium-concentration *R. palustris* (MRPRF), and high-concentration *R. palustris* (HRPRF) experimental groups received the basal diet supplemented with 20.0 mL, 40.0 mL, and 80.0 mL (concentration of 1 × 10^9^ CFU/mL) of *R. palustris* solution per goat per day, respectively, administered via feed mixing. The experimental period was 75 days. The basal diet was formulated according to Nutritional Requirements for Meat Sheep (NY/T816-2021) [27], and its composition is shown in Table 1.

### 2.3. Preparation of R. palustris

Expand the commercial *R. palustris* stock solution using photosynthetic bacteria culture medium (both purchased from Bainuo Biotechnology Co., Ltd., Yancheng, China). Prepare the liquid medium by mixing the solid medium with purified water at a 1:100 ratio. Then, combine the stock solution with the liquid medium at a ratio of 1~1.5:5 to create the *R. palustris* bacterial suspension for expansion. After aliquoting the prepared bacterial solution, place it under conditions with abundant sunlight and a suitable temperature (25–35 °C) for expansion. The optimal expansion period is 4–5 days. During this time, shake the liquid evenly to ensure uniform light exposure (supplement with 60–100-watt bulbs at night or on cloudy days) to enhance the expansion rate and biomass.

### 2.4. Sample Collection

On the 75th day of the trial, prior to the morning feeding, three goats were randomly selected from both the control and experimental groups and euthanized via jugular vein exsanguination. The abdominal cavity was promptly opened to expose the rumen. A small incision was made in the dorsal sac of the rumen, avoiding major blood vessels, and approximately 200 g of rumen content was collected. The content was immediately filtered through sterile gauze and aliquoted into 50 mL sterile centrifuge tubes for rumen microbiota diversity analysis. Samples were rapidly frozen in liquid nitrogen and subsequently stored in an ultra-low temperature freezer at −80 °C for preservation.

### 2.5. DNA Extraction, PCR Amplification, and Illumina MiSeq Sequencing

Total DNA of rumen microorganisms was extracted from frozen rumen samples using the E.Z.N.A.^®^ Soil DNA Kit (Omega Bio-tek, Norcross, GA, USA) according to the instructions, and the DNA concentration and purity were measured using a NanoDrop 2000 spectrophotometer (Thermo Fisher Scientific, Waltham, MA, USA), with the OD/OD_280_ ratio between 1.8 and 2.0 and the OD/OD_80_ ratio between 1.8 and 2.0. The OD_260_/OD_280_ ratio was required to be between 1.8 and 2.0, and the OD_260_/OD_230_ ratio was required to be greater than 2.0. The qualified DNA samples were diluted to 2.0 °C. The qualified DNA samples were diluted to 50 ng/μL and stored at −20 °C. The extracted DNA was used as a template to amplify and detect the full-length variable region (V1-V9) of the bacterial 16S rRNA gene, using primers 27F (AGRGTTTGATYNTGGCTCAG) and 1492R (TASGGHTACCTTGTTASGACTT). The PCR system was 25 μL, including 2 × Taq PCR Master Mix 12.5 μL, 0.5 μL each of upstream and downstream primers (10 μmol/L), 1 μL template DNA, and 10.5 μL ddH_2_O. The reaction conditions were as follows: 95 °C pre-denaturation, 0.5 μL each of upstream and downstream primers, 1 μL template DNA, 10.5 μL ddH_2_O, and 10.5 μL ddH_2_O. The reaction conditions were as follows: pre-denaturation at 95 °C for 3 min, denaturation at 95 °C for 30 s, annealing at 55 °C for 30 s, extension at 72 °C for 30 s for 30 cycles, and final extension at 72 °C for 10 min. The amplified products were detected by electrophoresis on a 1.5% agarose gel, and the target fragment was recovered by cutting the gel. The recovered PCR products were sent to a professional sequencing company (e.g., UW Genetics) for double-end sequencing (PE 300) on the Illumina MiSeq platform, and sequencing libraries were constructed and sequenced according to standard procedures.

### 2.6. Statistical Analysis

**Figure 9:**
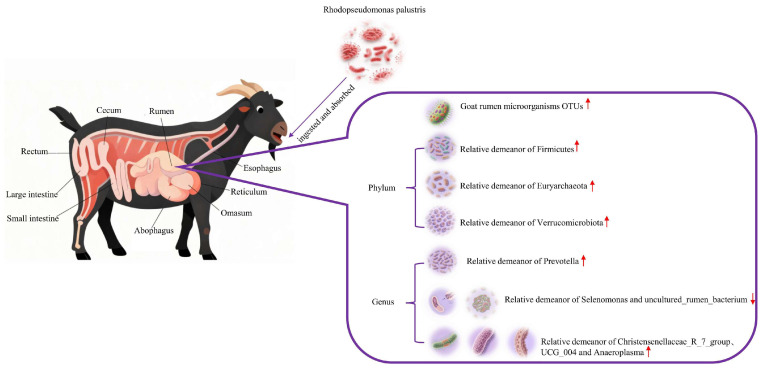
The changes in rumen microflora and diversity that occurred after the ingestion and uptake of *R. palustris* in Leizhou goats in terms of OTUs and the distribution of the flora at the phylum level and the genus level, respectively, were used to determine whether this organism positively regulated the rumen microflora of Leizhou goats (doubao version 1.76.3_win).

This experiment employed a randomized complete block design (RCBD). Goats of similar weight and age were randomly assigned to different treatment groups: control, supplemented with photosynthetic bacterial culture medium, and added low, medium, and high concentrations of *R. palustris*. The experimental data were organized using Microsoft Excel 2016. SPSS 25.0 software was employed for data analysis. One-way analysis of variance (ANOVA) was used to investigate the effects of different treatment groups on α-diversity indices and microbial abundance at the phylum and genus levels. When ANOVA detected significant differences, Duncan’s multiple range test was applied for post hoc comparisons among treatment groups. The results are presented as mean ± SD. *p* < 0.05 indicates significant differences, while 0.05 ≤ *p* < 0.10 indicates a non-significant trend. Part of Figure 9 utilizes GenAI tools (doubao version 1.76.3_win) to generate relevant illustrations of goats and fungal strains.

Sequencing data were quality controlled and analyzed using QIIME 2 (v2020.8) software. First, the original bipartite sequencing data were spliced and filtered for low-quality sequences, and chimeras were removed to obtain high-quality, valid sequences. Then, the effective sequences were clustered with 97% similarity to obtain operational taxonomic units (OTUs). Based on the OTUs, the Chao1 abundance index, the Shannon diversity index, and the Simpson dominance index were calculated to analyze the abundance and diversity of the rumen flora. Principal coordinate analysis (PCoA) and analysis of differences between groups (ANOSIM) were performed using the R language (v4.0.3) to explore the differences in rumen flora structure between the two groups of goats. LEfSe (LDA Effect Size) analysis was used to screen species with significant differences (LDA value ≥ 4.0, *p* < 0.05) between groups, and evolutionary branching diagrams and bar charts were drawn to show the distribution of differential species.

## 3. Results

### 3.1. Analysis of Microbial OTUs in Goat Rumen

**Figure 1:**
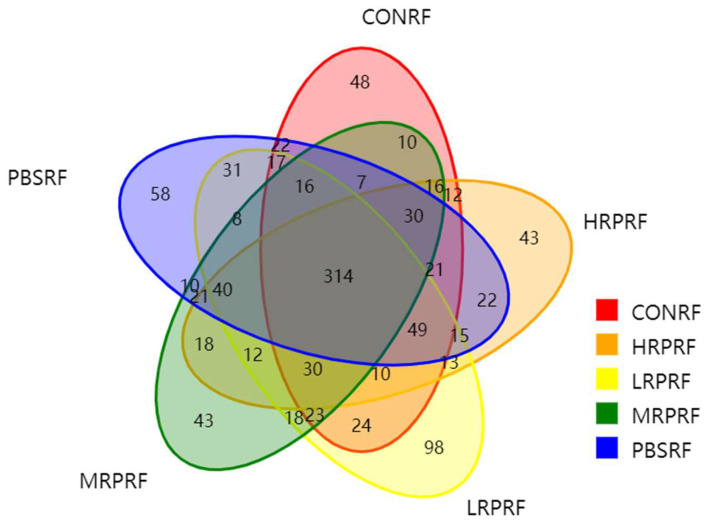
Venn diagram showing the number of operational taxonomic units (OTUs). CON = feeding only the basal diet; PBM (PBS) = basic diet and photosynthetic bacteria culture medium liquid; LRP = base diet supplemented with low *R. palustris*; MRP = base diet supplemented with medium *R. palustris*; HRP = base diet supplemented with high *R. palustris*; RF = rumen fluid.

As shown in Figure 1, a total of 1099 operational taxonomic units (OTUs) were detected, comprising 649 OTUs in the CONRF group, 681 in the PBMRF group, 718 in the LRPRF group, 616 in the MRPRF group, and 666 in the HRPRF group. Shared OTUs numbered 314, accounting for 28.57% of the total OTUs. The number of OTUs unique to the CONRF, PBMRF, LRPRF, MRPRF, and HRPRF groups was 48, 58, 98, 43, and 43, respectively. The order of OTU abundance was LRPRF group > PBMRF group > CONRF group > MRPRF group and HRPRF group. A comprehensive analysis revealed that both the total OTU count and the unique OTU count in the LRPRF group exceeded those in the CONRF group and other experimental groups. This indicates that supplementing the diet with *R. palustris* at a concentration of 20 mL/head increases rumen-specific microbial diversity.

### 3.2. Effect of R. palustris on the Alpha Diversity of Microorganisms in the Gastric Rumen of Goats

**Figure 2:**
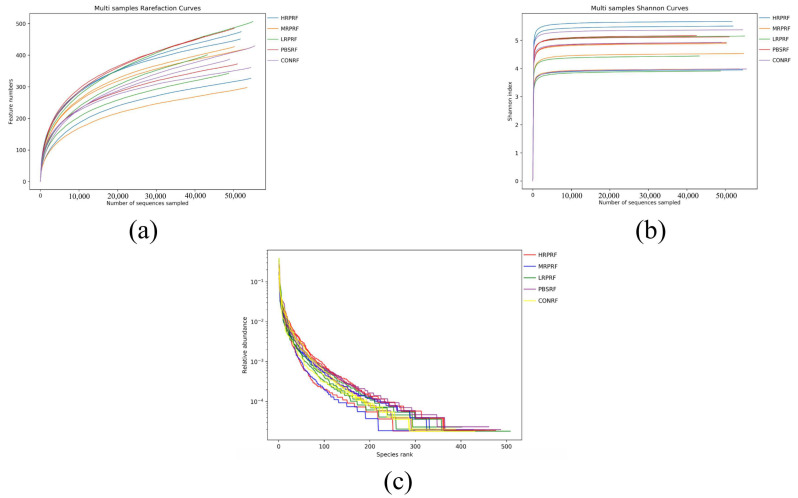
Alpha diversity analysis of rumen microorganisms. (**a**) The number of features was observed to vary with sequencing depth (rarefaction curve). (**b**) It was observed that more species were found as the sequencing volume increased until the species volume saturated (Shannon index). (**c**) Relative distribution of species abundance (rank abundance curve). CON = feeding only the basal diet; PBM (PBS) = basic diet and photosynthetic bacteria culture medium liquid; LRP = base diet supplemented with low *R. palustris*; MRP = base diet supplemented with medium *R. palustris*; HRP = base diet supplemented with high *R. palustris*; RF = rumen fluid.

Within a certain sequencing range, a steep increase in the rarefaction curve indicates the discovery of many new species, whereas a plateau suggests that additional sequencing yields few new species. In this study, the rarefaction curves (Figure 2a) showed a gradual increase with sequencing depth, without evident plateauing, indicating that new species continued to emerge, but the sequencing depth was sufficient to capture the majority of characteristic species. The Shannon index curve (Figure 2b) rose sharply at low sequencing depth and gradually approached saturation near 10,000 reads, suggesting that the sequencing volume was adequate to reliably assess rumen bacterial diversity. Rank-abundance curves (Figure 2c) were relatively broad and flat across all treatment groups, reflecting both high community richness and evenness. Overall, these results demonstrate that the sequencing depth was sufficient and that all treatment groups exhibited diverse and balanced rumen microbial communities.

**Figure 3:**
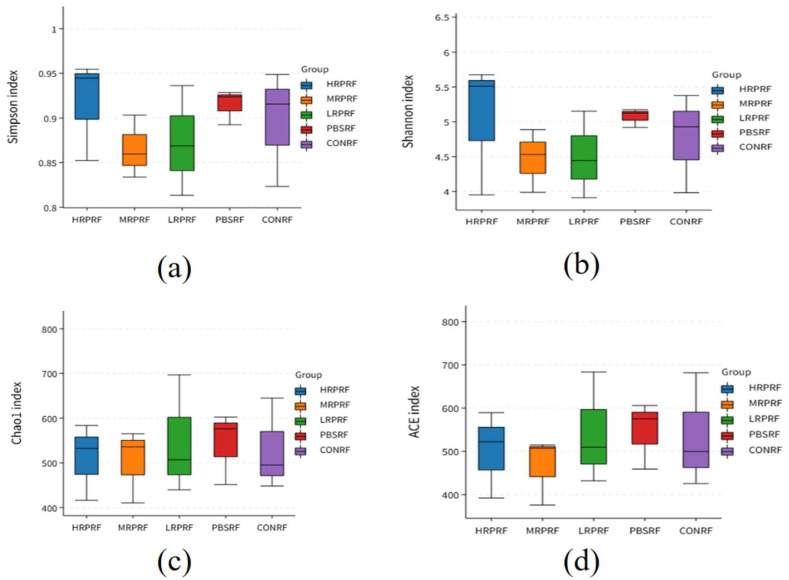
In microbiome research, alpha diversity serves as a core metric for assessing both richness and evenness within individual samples. This box plot focuses on five treatment groups: HRPRF, MRPRF, LRPRF, PBMRF, and CONRF. Using four key indices, Simpson, Shannon, Chao1, and ACE, it systematically reveals the effects of different treatments on the internal diversity of microbial communities. (**a**) Simpson index boxplots for different grouped samples, illustrating community dominance patterns; (**b**) Boxplots of the Shannon index for different grouped samples, illustrating community diversity; (**c**) Boxplots of the Chao1 index for different grouped samples, illustrating community richness; (**d**) Boxplots of the ACE index for different grouped samples, illustrating community diversity. CON = feeding only the basal diet; PBM (PBS) = basic diet and photosynthetic bacteria culture medium liquid; LRP = base diet supplemented with low *R. palustris*; MRP = base diet supplemented with medium *R. palustris*; HRP = base diet supplemented with high *R. palustris*; RF = rumen fluid.

As shown in Table 2, the coverage index exceeded 99% across all groups, indicating that the sequencing data comprehensively covered the majority of species information within the samples. Compared to the CONRF group, the PBSRF and LRPRF groups showed a trend toward an increased Ace index, while the MRPRF and HRPRF groups exhibited a similar trend for the Chao1 index. Similarly, the phylogenetic diversity (PD) whole tree index values increased across these groups. However, as depicted in Figure 3, no significant differences (*p* > 0.05) were observed between groups for Simpson indices, Shannon indices, Chao1 indices, or Ace indices.

### 3.3. Effect of R. palustris on the Diversity of Microorganisms in the Gastric Rumen of Goats

**Figure 4:**
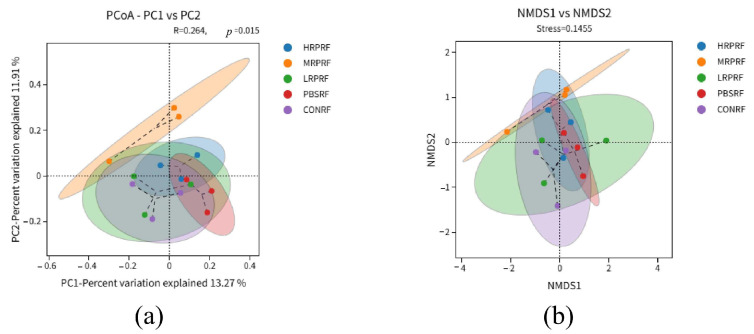
Beta diversity analysis of goat rumen coordinates and metric multidimensional calibration analysis. (**a**) Principal Coordinate Analysis (PCoA) of microbial communities. (**b**) Non-metric multidimensional scaling (NMDS) of microbial communities. CON = feeding only the basal diet; PBM (PBS) = basic diet and photosynthetic bacteria culture medium liquid; LRP = base diet supplemented with low *R. palustris*; MRP = base diet supplemented with medium *R. palustris*; HRP = base diet supplemented with high *R. palustris*; RF = rumen fluid.

Beta diversity refers to the variation in species composition between different communities along an environmental gradient, with greater distances between samples indicating greater divergence. PCoA analysis visually displays the bacterial community differences between control and experimental samples. The Bray–Jaccard distance algorithm was employed, and ANOSIM analysis was used for intergroup difference testing. PCoA analysis revealed a significant grouping effect (R^2^ = 0.264, *p* = 0.015), indicating that 13.27% and 11.91% of the variance were explained by PC1 and PC2, respectively, with statistically significant differences between groups (Figure 4a). NMDS analysis revealed differences in species composition between the control and experimental groups. A stress value of 0.1573 indicates reliable dimensionality reduction in the NMDS analysis, with the plot effectively reflecting differences in the original data (Figure 4b). Both analytical methods consistently revealed a distinct clustering tendency among samples from different treatment groups, confirming significant differences in microbial community structure between the HPPRF, MRPRF, LRPRF, PBSRF, and CONRF groups.

### 3.4. Effects of R. palustris on the Composition and Community Structure of Rumen Flora in Leizhou Goats

**Figure 5:**
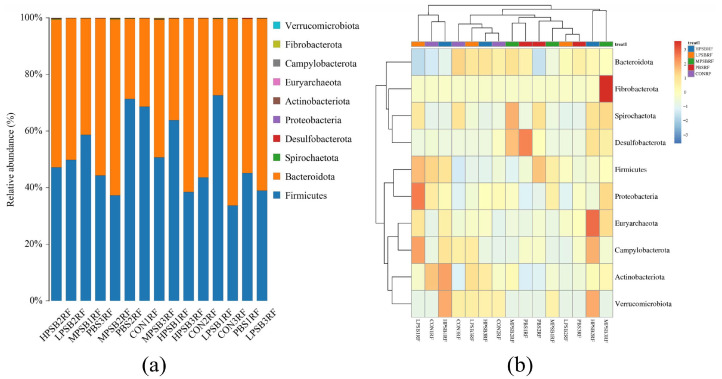
Rumen microbial composition at the phylum level. (**a**) Bar chart displaying taxonomic abundance. (**b**) Clustered heatmap of abundance profiles. CON = feeding only the basal diet; PBM (PBS) = basic diet and photosynthetic bacteria culture medium liquid; LRP = base diet supplemented with low *R. palustris*; MRP = base diet supplemented with medium *R. palustris*; HRP = base diet supplemented with high *R. palustris*; RF = rumen fluid.

As shown in Figure 5a,b, a total of 10 phyla were detected at the phylum level, including Firmicutes, Bacteroidota, Spirochaetota Desulfobacterota, Proteobacteria, Proteobacteria, and Actinobacteriota. As can be seen from Table 3, the rumen flora of each group of bacterial phylum was dominated by Firmicutes and Bacteroidetes at the phylum level, which accounted for more than 90% of the total bacterial structure. The relative abundance of Firmicutes and Bacteroidetes in the RPRF group, although numerically higher than that in the CONRF group, showed no statistically significant difference compared with the control group (*p* = 0.98). Specifically, compared with the CONRF and PBMRF groups, the relative abundance of Verrucomicrobiota in the *R. palustris* group was significantly higher than that in the CONRF and PBMRF groups (*p* = 0.02).

**Figure 6:**
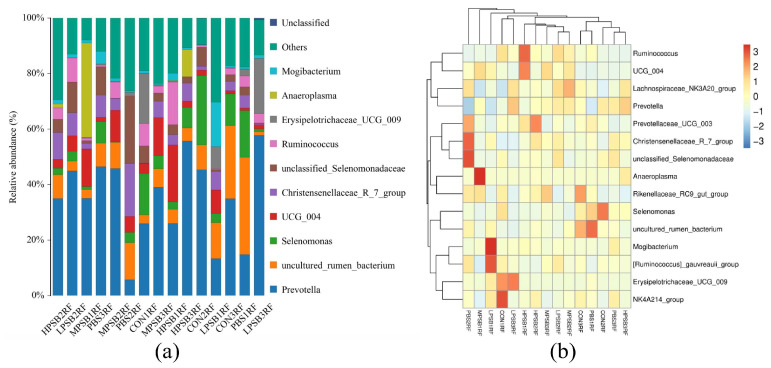
Relative abundance of rumen at the genus level. (**a**) Bar chart of species abundance at the genus level. (**b**) Cluster heatmap of genus-level abundance. CON = feeding only the basal diet; PBM (PBS) = basic diet and photosynthetic bacteria culture medium liquid; LRP = base diet supplemented with low *R. palustris*; MRP = base diet supplemented with medium *R. palustris*; HRP = base diet supplemented with high *R. palustris*; RF = rumen fluid.

As shown in Figure 6a,b, at the genus level, the major dominant bacteria included *Prevotella*, *uncultured rumen bacterium*, *Selenomonas*, and an *unclassified group (UCG-OO4)*. The main dominant bacteria include *Prevotella*, *uncultured_rumen_bacterium*, *Selenomonas*, *UCG-004*, *Christensenellaceae R-7 group*, *unclassified Selenomonadaceae*, and *Ruminococcus*. As can be seen from Table 4, the secondary dominant bacteria included *Erysipelotrichaceae UCG 009*, *Anaeroplasma*, *Mogibacterium*, and *Prevotellaceae UCG 003*, among others. Compared with the CONRF group, the relative abundance of *Selenomonas* in the four experimental groups was highly significantly lower (*p* < 0.05); the relative abundance of the *uncultured rumen bacteria* was lower (*p* > 0.05), but the relative abundance of the *Christensenellaceae R-7 group* was relatively higher (*p* > 0.05) in the RPRF groups. Compared to the CONRF group, PBMRF group, and LRPRF group, the MRPRF group and HRPRF group showed a trend toward relatively increased relative abundance of *Anaeroplasma* and *UCG-004*.

### 3.5. LEfSe Species Difference Analysis

**Figure 7:**
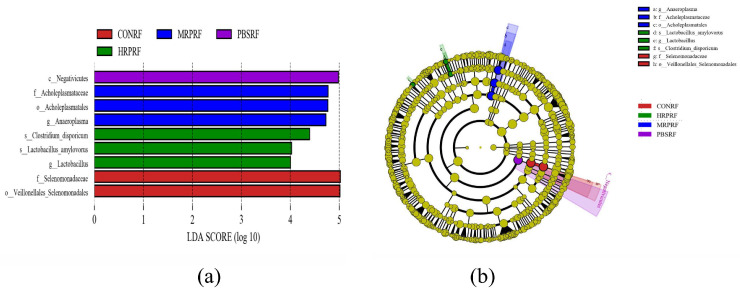
Display LDA scores between different groups, where LDA > 4.0 and *p* < 0.05. (**a**) The figure displays biomarkers with LDA scores exceeding the threshold value (LDA > 4.0), indicating statistically significant differences between groups. (**b**) In the evolutionary tree diagram, concentric circles radiating outward represent taxonomic levels from kingdom (single circle) to genus (or species). CON = feeding only the basal diet; PBM (PBS) = basic diet and photosynthetic bacteria culture medium liquid; LRP = base diet supplemented with low *R. palustris*; MRP = base diet supplemented with medium *R. palustris*; HRP = base diet supplemented with high *R. palustris*; RF = rumen fluid.

As shown in Figure 7a, after screening with a linear discriminant analysis (LDA) threshold greater than 4, the main bacterial groups exhibiting significant differences in abundance in the rumen included *f_Acholeplasmataceae*, *f_Selenomonadaceae*, *s_Clostridium_disporicum*, and nine other groups. Compared to the CONRF group and the PBMRF group, the MRPRF and HRPRF groups had a higher number of significantly different bacterial groups. As shown in Figure 7b, the key microbial groups in the CONRF group were *o_Vellionellales_Selenomonadales* and *f_Selenomonadaceae*. In contrast, the key microbial groups in the MRPRF and HRPRF groups were *g_Anaeroplasma* and *s_Lactobacillus amylovorus*, among others. Compared to the CONRF group, the MRPRF and HRPRF groups exhibited a higher number of significantly contributing microbial groups.

### 3.6. Predictive Analysis of Colony Function (Picrust2)

**Figure 8:**
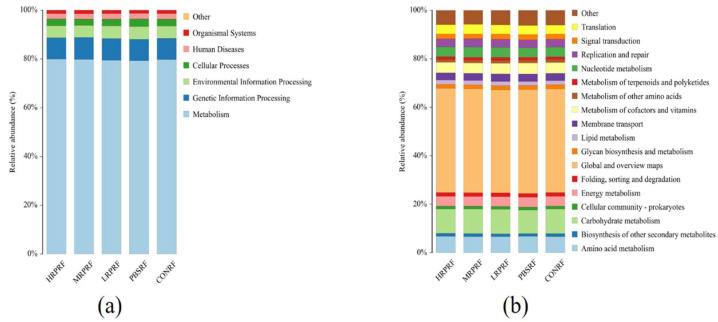
Functional analysis of rumen microorganisms. (**a**) Distribution of relative abundance of level 1 in Picrust2 functional prediction across different samples; (**b**) Distribution of relative abundance of level 2 in Picrust2 functional prediction across different samples. CON = feeding only the basal diet; PBM (PBS) = basic diet and photosynthetic bacteria culture medium liquid; LRP = base diet supplemented with low *R. palustris*; MRP = base diet supplemented with medium *R. palustris*; HRP = base diet supplemented with high *R. palustris*; RF = rumen fluid.

As can be seen from Figure 8a, rumen microbial functional genes were mainly categorized as metabolic pathways. As can be seen from Figure 8b, the relative abundance of rumen microbial functional genes from high to low were global and overview mapping, carbohydrate metabolism, amino acid metabolism, metabolism of cofactors and vitamins, and nucleotide metabolism, respectively. It can be concluded that there are no significant differences in functional genes between different groups.

## 4. Discussion

High-throughput sequencing has been widely used to study the microbiota in ruminants as a rapid and efficient method to determine the structure of rumen microbial communities [28]. Therefore, high-throughput sequencing methods can be used to show the overall effect of *R. palustris* in regulating the rumen microbiota of Leizhou goats. In this study, the sparse curves of the samples analyzed by high-throughput sequencing were relatively flat. This indicates that the data of our study are reasonable.

### 4.1. Influence of R. palustris on Rumen Microbial Diversity

As the largest compartment of the ruminant digestive system, the rumen is compartmentalized into various sacs. This environment sustains one of the most spectrally diverse ecosystems in nature, harboring a complex microbial community that includes a wide spectrum of bacteria, archaea, fungi, and ciliated protozoa. These microbial populations orchestrate the primary degradation of dietary plant polymers into monomers, culminating in the production of volatile fatty acids that supply the ruminant’s carbon and energy needs [29]. YY Chen et al. [24] found in an in vitro rumen fermentation assay that the addition of *R. palustris* improved the viability of rumen microorganisms, thereby promoting microbial fermentation, with high potential to promote rumen microbial growth and enhance the supply of microbial fermentation to the energy of the non-biogenic sugars through the maintenance of an anaerobic environment for microbial equilibrium. Consistent with the results of this study, the addition of *R. palustris* increased the number of OUTs as well as unique OUTs in the rumen of Leizhou goats.

Species richness (estimated by the Ace and Chao indices) and community diversity (assessed by the Shannon and Simpson indices) were evaluated. It is noteworthy that elevated values of the Shannon and Simpson indices are indicative of higher community diversity. Alpha diversity analysis showed that the Ace, Chao1, and PD whole tree indices of the experimental group were higher than those of the control group, and although the differences between the groups were not significant (*p* > 0.05), there was a trend of increase, suggesting that *R. palustris* may optimize the abundance and phylogenetic diversity of microorganisms by enhancing the rumen function. In addition, the Shannon and Simpson indices of the experimental group were slightly higher than those of the control group, further confirming the positive effect of *R. palustris* on microbial community evenness.

### 4.2. Influence of R. palustris on the Structure of Rumen Microbial Communities

B ADehority et al. [30] isolated 44 bacterial strains from the rumen contents of goats, of which *Vibrio butyricus* accounted for about 70% of the overall isolates, and *Vibrio butyricus* belongs to the *Firmicutes* phylum *Trichoderma*. Many studies have also shown that [31,32], at the phylum level, the most numerically dominant of the terrestrial mammalian gut microbiomes are the *Firmicutes* and the *Bacteroidota*. Dan Xue et al. [33] found that the dominant phyla in the rumen microorganisms during the growth phase of ruminants were *Firmicutes* phylum and *anaplasma* phylum, and the results of the present study were consistent with them. The *Firmicutes* phylum is a key group of rumen microorganisms in the process of roughage utilization, which is mainly involved in the degradation and utilization of cellulose and hemicellulose. *Firmicutes* can increase the abundance of genes encoding enzymes related to energy metabolism, promote the digestibility of oligosaccharides, starch, and cellulose, and improve enzyme activity and rumen fermentation [34,35]. *Bacteroidota* is mainly involved in the decomposition and absorption of non-fiber plant components during rumen fermentation in ruminants [36]. It has been shown that an increase in the relative abundance of the *Firmicutes* and a decrease in the relative abundance of the *Bacteroidota* can promote the growth of goats [37]. The results of this experiment showed that the addition of *R. palustris* increased the relative abundance of *Firmicutes* and decreased the relative abundance of *Bacteroidota* in the rumen of Leizhou goats, thus promoting the digestion and absorption of roughage in goats. In this experiment, dietary addition of *R. palustris* increased the relative abundance of the *Euryarchaeota* as well as significantly increased the relative abundance of *Verrucomicrobiota* (*p* < 0.05). *Archaea* comprised only 3–4% of the rumen microbiome, whereas the *Euryarchaea* were the dominant archaea in the rumen [38]. It has been shown that [39], in the rumen fluid of goats, *Euryarchaeota* accounts for 82% of the composition of methanogenic bacteria. Li et al. [40] found that the *Euryarchaeota* were positively correlated with body weight; Broad Archaea accounted for a higher percentage of the obese group, and it may be one of the strongest predictors of obesity measures [41]. Vera Guerra [42], in a study of the microbial community of the jejunum of grazing goats, found that *Verrucomicrobiota* and *Bacillota,* among others, belonged to the dominant flora. Some studies have claimed that [43,44] the *Verrucomicrobiota* is involved in the degradation of intestinal polysaccharides, such as fibrous disaccharides, etc. *Verrucomicrobiota* are considered highly suited for lignocellulose degradation in the rumen because they can encode various carbohydrate-degrading enzymes, peptidases, and sulfatases [45]. Concurrently, a metagenomic study has also revealed their unique role in rumen lignocellulose degradation [46]. Bamola mentioned in the report that [47] the reduced abundance of *Verrucomicrobiota* may potentially affect the preservation of intestinal mucosal barrier and immunomodulatory functions. In this study, the increased relative abundance of *Verrucomicrobiota* suggests that *R. palustris* holds potential for improving ruminal degradation of lignocellulose and enhancing the intestinal immune barrier.

At the genus level, one of the major carbohydrate-degrading microorganisms, *Prevotella*, had the highest relative abundance [48,49], consistent with the results of this study. Bekele et al. found that [50] *Prevotella* accounted for 56% or 60% of the rumen bacteria in goats. The results of the study showed that the addition of *R. palustris* to the ration increased the relative abundance of *Prevotella* in the rumen flora. The main function of *Prevotella* is protein degradation, but it is also involved in fiber degradation [51,52]. *Prevotella* is abundant in many ruminants; one of its fermentation products is propionic acid, and thus it has the potential to compete with *methanogenic* and *archaeal bacteria* for hydrogen utilization. Its abundance is negatively correlated with methane emissions, and *Prevotella* may have potential for use as an antimethanogenic agent [53]. *Selenomonas* crescentus plays a critical role in the rumen as a key consumer of lactic acid. Reducing lactic acid accumulation helps stabilize ruminal pH and creates a more favorable environment for other rumen microorganisms. Furthermore, this bacterium promotes the synthesis of propionic acid, thereby enhancing overall rumen fermentation efficiency [54]. It has been shown that [55] lipopolysaccharide in the rumen is positively correlated with *Selenomonas*. Douglas B. Jordan et al. [56] found in ruminant studies that *Selenomonas* can produce β-d-xylosidase in animals. *Uncultured rumen bacterium* may play a role in rice straw digestion [57]. In prior studies [58], it has been shown that most fiber-associated bacterial communities are uncultured bacteria. It has been suggested that [59] the role of *uncultured rumen bacterium* in the rumen of ruminants may be responsible for fiber digestion in the rumen. K. N. Joblin mentioned that [60] all rumen mycoplasmas found so far have been categorized in the genus *Astroplasma* or *anaerobic protozoa*, which were produced to adapt to rumen mycoplasmas. The interaction between the gut microbiota and the gastrointestinal immune system is of particular importance for animal health, and important elements of the gastrointestinal immune response are the cytokines TGF-β and IgA antibodies. TGF-β is a potent immunomodulatory cytokine that suppresses adverse immune responses in the gut and induces IgA-like exchanges. Bacteria belonging to the *Anaeroplasma*, on the other hand, have the ability to enhance the level of mucosal IgA and induce the synthesis of TGF-β, a key regulatory cytokine, in T cells. *Anaeroplasma* categorized the anti-inflammatory cytokine TGF-β also fortifies the intestinal barrier by promoting mucosal IgA production [61,62]. *Selenomonas* is a functionally diverse and important bacterium in the rumen, exhibiting strong adaptability and the ability to survive under extreme nutritional fluctuations. Its primary function is not the direct degradation of complex polysaccharides, such as dietary fiber, but rather the efficient utilization of soluble carbohydrates produced by other bacteria through hydrolysis [63]. A study indicates that [64] *Selenomonas* may participate in and stimulate the digestion of rumen fiber, with increased propionate production. However, Schingoethe’s report indicates that [65] *Selenomonas* bacteria in the rumen of ruminants possess the ability to ferment starch and release lactic acid within the rumen, thereby increasing the risk of low rumen pH. In this study, the relative abundance of genus-level *Selenomonas* decreased, potentially weakening the rumen’s capacity to degrade carbohydrates and lignocellulose. However, this reduction also balanced the pH within the rumen environment, thereby reducing the risk of rumen acidosis caused by low pH levels.

### 4.3. Influence of R. palustris on Rumen Differential Flora

LEfSe analysis (Figure 7) showed that the number of differential flora in the test group was more than that in the CONRF group; especially, the MRPRF group had more significant differential flora, including *Prevotella*, *g-Lactobacillus*, and *Selenomonadaceae*. Alterations in these flora may directly affect rumen fermentation patterns and host metabolism. For example, *Lactobacillus* may stimulate an increase in the innate immune response; the use of *Lactobacillus* in food is widely distributed, and it has been studied for its control of intestinal infections, effects on cholesterol levels, and anticancer effects [66,67,68,69]. V Chiofalo et al. [70] found in growth trials with lambs that the addition of *Lactobacillus rhamnosus* positively affected the development and maintenance of fermenting rumen activity, increasing feed utilization and dry matter intake; determined optimal pH conditions for pancreatic enzyme activity, which improved intestinal absorption of nutrients; and reduced protein-hydrolyzing microorganisms, which improved the nutritive properties of the intestinal mucosa in a secreted and absorbed manner. *Lactobacillus rhamnosus* strains isolated from goat’s milk can act as immunoactive modulators of intestinal and respiratory infections [71]. In vivo, *G-Lactobacillus* regulates the intestinal flora, thereby maintaining its homeostasis. It also protects against pathogenic bacteria by competing for adhesion sites [72], and *Lactobacillus* spp. can inhibit the proliferation of pathogenic bacteria in the rumen by producing organic acids and antimicrobial peptides [73,74]. Lactic acid bacteria (LAB) produce various antimicrobial compounds—including lactic acid, short-chain fatty acids, hydrogen peroxide, and bacteriocin-like substances—that inhibit the growth of pathogens. Furthermore, they enhance immune function and lower the susceptibility to allergic responses [75], whereas the enrichment of certain spore-producing bacteria (e.g., *Turicibacter*) may be associated with fat deposition. In a previous report [76], *Turicibacter* emerged as a key regulator of host adipose biology, whose colonization effectively modulated host lipid metabolism, leading to reduced serum triglycerides and adipose tissue mass. However, Liu et al. [77] indicated in their study that *Turicibacter* may be one of the elements that cause the inflammation that occurs in the epithelium of the hindgut mucosa.

### 4.4. Changes in the Environment of the Rumen

As shown in Figure 9, in the rumen microbial ecosystem of Leizhou goats, introducing *R. palustris* may optimize rumen fermentation function and promote microbial community health. Specifically, increased relative abundance of *Firmicutes* and *Euryarchaeota* indicates enhanced carbohydrate degradation capacity and methane metabolic activity, potentially improving feed energy utilization efficiency; increased abundance of *Verrucomicrobiota* and *Prevotella* suggests enhanced fiber degradation and protein metabolism processes, which are beneficial for roughage digestion and absorption. The enrichment of beneficial microorganisms, such as *Christensenellaceae_R-7_group*, further supports the trend toward a stable and healthy rumen microbial community structure; conversely, the reduced abundance of bacterial groups, like *Selenomonas,* may reflect an overall optimization of microbial metabolic functions toward greater efficiency.

## 5. Conclusions

Research indicates that supplementing the diet of Leizhou goats with low concentrations of *R. palustris* can increase the number of unique OTUs within their rumen microbiota. At the phylum level, Firmicutes and Bacteroidota were identified as the dominant bacterial phyla in the rumen of Leizhou goats. Compared with the CONRF group, the relative abundances of Verrucomicrobiota and Euryarchaeota in the *R. palustris*-supplemented groups showed a significant increase. At the genus level, the main dominant genera were *Prevotella*, uncultured rumen bacterium, and *Selenomonas*. Specifically, the relative abundance of *Selenomonas* was decreased, and that of the uncultured rumen bacterium was significantly decreased in the supplemented groups. In contrast, the relative abundances of the *Christensenellaceae R-7 group*, *Anaeroplasma*, and *UCG-004* were increased, though the differences did not reach statistical significance. Furthermore, LEfSe analysis of species-level differences revealed that *R. palustris* also exerted a positive regulatory effect on the relative abundances of rumen microorganisms, such as *Prevotella*, *g-Lactobacillus*, and *Selenomonadaceae*, further confirming its role in optimizing rumen microbial community structure.

## Figures and Tables

**Table 1:** Basic feed composition and nutritional levels (as-fed basis).

Items	Content %
Pennisetum × sinese	56.00
Corn	22.00
Soybean meal	11.00
Bran (*Triticum aestivum* L.)	4.50
Cottonseed meal	4.50
Limestone powder	0.30
Calcium hydrogen phosphate	0.20
Salt	0.30
Compound premix ^1^	1.20
Total	100.00
**Nutritional level**	**Content**
ME MJ/kg ^2^	9.80
DM (%)	56.06
CP (%)	16.20
NDF (%)	45.50
ADF (%)	30.20
Ca (%)	0.92
P (%)	0.51

^1^ Per kilogram of diet, the premix provides the following: 8000 IU of vitamin A, 50 IU of vitamin E, 1200 IU of vitamin D3, 2000 mg of L-lysine, 1500 mg of DL-methionine, 50 mg of iron, 15 mg of copper, 50 mg of zinc, 50 mg of manganese, 10.5 mg of iodine, 0.3 mg of cobalt, 150 mg of Ethoxyquinoline, and 5000 mg of lightweight calcium carbonate. ^2^ Metabolizable energy is calculated according to NY/T816-2021, and the rest of the values are measured.

**Table 2:** Alpha diversity index.

Items	CONRF Group	PBMRF Group	LRPRF Group	MRPRF Group	HRPRF Group	*p*-Value
Ace index	533.78 ± 131.49	546.80 ± 77.32	541.84 ± 128.52	466.23 ± 77.89	501.44 ± 100.24	0.863
Chao1 index	529.46 ± 102.62	510.66 ± 85.66	503.98 ± 133.19	547.92 ± 133.19	543.30 ± 80.62	0.974
Simpson index	0.89 ± 0.06	0.91 ± 0.05	0.86 ± 0.03	0.87 ± 0.06	0.91 ± 0.01	0.637
Shannon index	4.76 ± 0.71	5.04 ± 0.95	4.46 ± 0.45	4.50 ± 0.62	5.07 ± 0.13	0.665
PD whole tree	20.31 ± 2.89	21.86 ± 3.19	21.19 ± 2.35	22.40 ± 2.79	22.27 ± 2.89	0.886
Coverage %	99.800	99.823	99.823	99.766	99.776	0.474

CON = feeding only the basal diet; PBM = basic diet and photosynthetic bacteria culture medium liquid; LRP = base diet supplemented with low *R. palustris*; MRP = base diet supplemented with medium *R. palustris*; HRP = base diet supplemented with high *R. palustris*. RF = rumen fluid.

**Table 3:** Effect of *R. palustris* on the relative abundance of rumen flora of Leizhou goats on the phylum.

Items	CONRF Group %	PBMRF Group %	LRPRF Group %	MRPRF Group %	HRPRF Group %	*p*-Value
Firmicutes	48.65 ± 17.99	53.61 ± 15.40	53.80 ± 17.21	48.91 ± 10.82	49.82 ± 12.89	0.98
Bacteroidota	51.17 ± 17.96	46.15 ± 15.44	46.00 ± 17.32	50.67 ± 10.66	49.89 ± 12.89	0.98
Spirochaetota	0.65 ± 0.79	0.68 ± 0.85	0.63 ± 0.69	1.45 ± 1.04	0.66 ± 0.87	0.72
Desulfobacterota	0.22 ± 0.19	1.08 ± 1.35	0.02 ± 0.03	0.99 ± 0.95	0.47 ± 0.82	0.48
Proteobacteria	0.37 ± 0.20	0.21 ± 0.11	0.28 ± 0.00	0.51 ± 0.01	0.36 ± 0.08	0.16
Actinobacteriota	0.31 ± 0.24	0.14 ± 0.06	0.31 ± 0.17	0.31 ± 0.06	0.47 ± 0.17	0.23
Euryarchaeota	0.04 ± 0.05	0.11 ± 0.07	0.21 ± 0.12	0.17 ± 0.21	0.38 ± 0.29	0.26
Campylobacterota	0.10 ± 0.14	0.11 ± 0.00	0.27 ± 0.27	0.04 ± 0.04	0.29 ± 0.23	0.36
Fibrobacterota	<0.01	<0.01	0.01 ± 0.02	0.64 ± 0.11	0.05 ± 0.04	0.47
Verrucomicrobiota	0.01 ± 0.01 ^a^	<0.01 ^a^	0.06 ± 0.01 ^b^	0.06 ± 0.01 ^b^	0.03 ± 0.01 ^b^	0.02

CON = feeding only the basal diet; PBM= basic diet and photosynthetic bacteria culture medium liquid; LRP = base diet supplemented with low *R. palustris*; MRP = base diet supplemented with medium *R. palustris*; HRP = base diet supplemented with high *R. palustris*. RF = rumen fluid. ^a,b^ within a row means that they do not share the same letter and are significantly different by Duncan’s multiple range test (*p* < 0.05).

**Table 4:** Effect of *R. palustris* on the relative abundance of rumen flora of Leizhou goats on the genus.

Items	CONRF Group %	PBMRF Group %	LRPRF Group %	MRPRF Group %	HRPRF Group %	*p*-Value
*Prevotella*	35.40 ± 9.74	22.33 ± 21.41	38.68 ± 22.84	39.97 ± 5.42	38.93 ± 15.24	0.67
*uncultured_rumen_bacterium*	12.83 ± 12.02	18.95 ± 14.13	5.91 ± 6.12	6.40 ± 3.15	6.11 ± 3.15	0.34
*Selenomonas*	17.11 ± 7.02 ^b^	9.47 ± 3.87 ^b^	2.63 ± 1.19 ^a^	2.04 ± 2.40 ^a^	4.12 ± 2.82 ^a^	0.01
*UCG_004*	2.20 ± 1.22	2.67 ± 2.67	5.16 ± 3.48	12.28 ± 1.28	8.67 ± 10.29	0.12
*Christensenellaceae_R_7_group*	1.63 ± 1.67	10.59 ± 7.52	5.19 ± 4.17	4.08 ± 1.94	6.59 ± 2.99	0.19
*unclassified_Selenomonadaceae*	5.14 ± 2.44	12.53 ± 10.88	3.89 ± 6.19	1.34 ± 1.46	3.54 ± 1.96	0.23
*Ruminococcus*	3.72 ± 3.80	1.56 ± 2.08	4.15 ± 4.22	3.06 ± 2.52	6.53 ± 7.91	0.76
*Erysipelotrichaceae_UCG_009*	6.13 ± 10.38	1.33 ± 1.02	9.32 ± 10.00	0.30 ± 0.08	0.10 ± 0.14	0.36
*Anaeroplasma*	0.01 ± 0.01	<0.01	<0.01	11.40 ± 19.56	3.81 ± 5.18	0.50
*Mogibacterium*	0.58 ± 0.41	1.92 ± 0.22	6.16 ± 0.85	0.85 ± 0.30	1.58 ± 0.98	0.46
*Prevotellaceae_UCG_003*	1.92 ± 0.14	3.12 ± 2.68	1.25 ± 0.67	0.89 ± 0.44	3.83 ± 2.84	0.28
*Lachnospiraceae_NK3A20_group*	0.75 ± 1.00	1.75 ± 0.96	2.29 ± 1.09	1.63 ± 1.86	1.79 ± 0.73	0.63
*[Ruminococcus]_gauvreauii_group*	1.47 ± 0.38	1.33 ± 0.93	2.56 ± 1.77	0.85 ± 0.65	0.75 ± 0.23	0.23
*NK4A214_group*	1.98 ± 1.90	1.24 ± 0.40	1.05 ± 0.76	0.43 ± 0.41	1.04 ± 0.48	0.46
Others	4.68 ± 1.03	6.51 ± 1.79	4.60 ± 2.73	5.82 ± 4.13	4.46 ± 1.66	0.81

CON = feeding only the basal diet; PBM = basic diet and photosynthetic bacteria culture medium liquid; LRP = base diet supplemented with low *R. palustris*; MRP = base diet supplemented with medium *R. palustris*; HRP = base diet supplemented with high *R. palustris*. RF = rumen fluid. ^a,b^ within a row means that they do not share the same letter and are significantly different by Duncan’s multiple range test (*p* < 0.05).

## Data Availability

The dataset is available upon request from the authors.
